# Cultural Transformation in Healthcare: How Well Does the Veterans Health Administration Vision for Whole Person Care Fit the Needs of Patients at an Academic Rehabilitation Center?

**DOI:** 10.1177/2164957X221082994

**Published:** 2022-03-17

**Authors:** Jessica L Barnhill, Isabel J Roth, Keturah R Faurot, Gilson D Honvoh, Chanee E Lynch, Karla L Thompson, Susan A Gaylord

**Affiliations:** 1 2332 University of North Carolina, Chapel Hill, NC, USA

**Keywords:** whole health, mind-body, health care, holistic care, integrative medicine, lifestyle, transformation

## Abstract

**Background:**

The Veterans Health Administration is undergoing a cultural transformation toward person-driven care referred to as the Whole Health System of Care.

**Objective:**

This pilot study evaluated whether the Whole Health model resonates with patients of a large public university rehabilitation clinic.

**Methods:**

Thirty participants completed the Veterans Health Administration’s Personal Health Inventory (PHI), and six attended the course “Taking Charge of My Life and Health.” Researchers analyzed PHI responses and post-course focus group transcripts. A short post-PHI survey and post-course evaluation were collected.

**Results:**

Participants agreed the PHI is a simple, useful tool. The course, while well attended, did not meet participants’ expectations. Participants wanted access to integrative therapies and opportunities to contribute to healthcare transformation.

**Conclusion:**

Rehabilitation patients resonated with the Whole Health vision. They expressed enthusiasm for the cultural transformation represented by the model along with frustration that standard healthcare experiences fall short of this vision.

## Introduction

Presented with a need to expand access to integrative health services, researchers hypothesized that the vision of Whole Health (WH), developed by the Veterans Health Administration (VA), could promote an expansive view of integrative health that supports human flourishing.^1,2^ This pilot study explored whether two WH materials, the Personal Health Inventory (PHI) and the course “Taking Charge of My Life and Health”^3^ resonated with patients at a large academic rehabilitation clinic, and whether these materials could be transposed onto a different clinical setting within another healthcare system.

## Background

In 2011, the VA established the Office of Patient Centered Care and Cultural Transformation to “transform the VA healthcare system from the traditional model of healthcare to a personalized, proactive, patient-driven model that focuses on developing and advancing Whole Health for Veterans and employees.”^4^ This transformation gathered additional urgency with the 2016 passage of the Comprehensive Addiction and Recovery Act mandating the VA provide non-pharmacologic pain treatments, such as complementary and integrative health (CIH), to all Veterans with chronic pain.^5^

Central to WH are principles of integrative health. These include wholeness and balance, promotion of self-care and self-healing abilities, and the incorporation of mind-body and other non-pharmacologic approaches in an individualized care plan.^6,7^

The structure of the VA differs significantly from non-VA academic medical centers. Differences include reimbursement methods, continuity of care, and care coordination. Therefore, researchers wondered if the WH model was a feasible and acceptable way to expand delivery of CIH services at an academic rehabilitation center.

## Methods

This mixed methods study design is primarily a qualitative study with synchronous collection of quantitative data (QUAL-quant).^8^ The study utilized 2 introductory components of WH: the PHI^9^ and the course “Taking Charge of My Life and Health.”^10^ The PHI is a personal health-planning tool that describes the WH framework and guides patients’ conversations about values and goals. “Taking Charge of My Life and Health” is a VA course that meets weekly for 10 weeks to explore WH components. Each week contains some proportion of group sharing, mindfulness instruction, and education emphasizing the development of personal health goals.^1^

### Target Population and Recruitment Methods

The target population was a cross-section of adult patients seen by physiatrists and allied health staff. Patients were recruited through flyers in the waiting room and encouragement from their providers. A convenience sample was recruited at times when researchers were on hand to meet patients at the end of their appointments. The study was advertised as an opportunity to help inform the development of integrative health services at the Center for Rehabilitation Care (CRC). Thirty participants completed the PHI and six chose to attend the course. Researchers delivered the PHI and the course as designed, without modifications. Researchers obtained Institutional Review Board approval and written informed consent.

### Measures

Measures included: (1) responses to the PHI; (2) post-PHI questionnaire; (3) course evaluation; (4) post-participation focus group; and (5) group attendance.

The PHI begins with two questions: “What REALLY matters to you in your life?” and “What brings you a sense of joy and happiness?” The tool then presents eight components of health: moving the body, surroundings, personal development, food and drink, recharge, relationships, spirit and soul, and power of the mind. Concluding questions are “Now that you have thought about all of these areas, what is your vision of your best possible health? What would your life look like? What kind of activities would you be doing?” and “Are there any areas you would like to work on? Where might you start?”

Researchers administered a short survey immediately upon completion of the PHI. Questions assessed whether participants thought the PHI was useful, and if they were likely to share the results with their provider.

Course attendance was recorded as a surrogate measure of acceptability and feasibility. Participants were asked to rate whether they would recommend the course to others. Two experienced qualitative researchers [IR and JB] conducted the focus group at the conclusion of the last class using a semi-structured interview guide. Participants were asked about their most and least favorite aspects of the course and about the usefulness, challenges, or recommended changes to the course. They were also asked, “How was the program useful for managing your health and wellbeing?”

### Analysis Methods

PHI responses and focus group data were coded using thematic content analysis.^11^ Qualitative responses to the PHI were used to evaluate the feasibility of the tool for health promotion. Four coders, including experienced qualitative researchers (JB, IR), first read all content in each document and focus group transcript. Upon rereading, they independently identified codes within and among documents. Codes were not pre-specified rather they emerged from documents and developed by discussion among researchers. Researchers then decided on final codes together and grouped them into themes.

## Results

### Demographics

Participants completing the PHI were predominantly female (79%). There were no Hispanic participants. Black/African American participants represented 28% of the sample and represent 22% of North Carolina’s population.^12^ Ages ranged from 26 to 74 years with a mean of 54 years. There was a broad distribution of insurance types. (see Table 1) Among the six group participants, five were female and one identified as “genderqueer.” One group participant was Black/African American and five were White/European-American.

**Table 1.** Participant Demographics.

Gender			(%)			N = 28		
Female			79			22		
Male			18			5		
Genderqueer			4			1		
Race								
Black or African American			29			8		
White or European American			68			19		
Other			4			1		
Insurance type								
None			14			4		
Medicaid only			4			1		
Medicare only			11			3		
Medicaid and Medicare			18			5		
Private			32			9		
Private + Medicare			18			5		
Veterans Administration			4			1		
Age			Mean			Range		
			54			26-74		

### Personal Health Inventory Themes

Using the PHI as a guide elicited substantive responses from patients related to meaning and purpose in their lives. Prominent themes included family/friends/pets; autonomy/safety/independence; meaningful connection/helping others; and God/church/spirituality.

### Post-PHI Survey

Patients understood the questions and were receptive to using the tool. When asked if they thought the PHI could help improve their health, eighty-three percent said “definitely yes” or “probably yes.” Seventy-two percent said that they would “definitely” share the PHI with their doctor, therapist, or provider. Eighty-six percent said they would “definitely” or “probably” join a Whole Health class.

### Course Evaluation

Course evaluations were mixed. When asked, “Would you recommend the course to family and friends?” One participant (14.3%) answered “definitely yes,” two (28.6%) answered “probably yes,” and three (57.1%) answered “maybe.”

### Focus Group Findings

All course members attended the focus group. The following themes emerged:

#### 1. Resonance With Model Accompanied by Frustration at Current State of Healthcare

The holistic model resonated with participants and its absence frustrated them. They wanted access, affordability, and integration of complementary modalities with conventional care.

“I would assume in actual practice there would be a better integration of the client with the program and matching them with all of the services that they need and of course, there is the issue you know of the funding and all that comes into play, which of course is a whole nother animal that we don’t want to talk about today. Because I don’t think we can address that.” – Participant three

“The challenges are really finding out about the resources and what is available to us whether we have insurance, no insurance, working, not working.” – Participant four

#### 2. While Participants Had Different Expectations for the Course, They Agreed That Their Expectations were Not Met

Most participants expressed that the course was not what they thought it would be. Some expected access to CIH treatments tailored to their symptom complaints, insurance status, and disposable income. Participants were looking for both active and passive CIH modalities, with some participants wanting further training in mind-body modalities and others expecting access to massage and acupuncture.

“What we’re asking for is other health providers whether it’s, you know your acupuncturist or massage therapist or whatever. Come in and help us guide us through some of this stuff. Help us, show us how to make this work better in our lives. Not just have us sit and think about it.” - Participant two

#### 3. Core Components of the Course, Such as Health Goals and Self-Management did Not Resonant With Participants

There was a noticeable lack of discussion in the focus group of core course materials. For example, content regarding health goals appeared often, but focus group members did not reference this.

#### 4. Despite Their Differing Expectations and Shared Frustration, They Kept Showing Up

There was near 100% participation in the course. Aside from select dates for which participants had previously noted conflicts, the six course members completed nearly all ten sessions. Some participants traveled long distances to attend the class. One participant described regularly waiting in traffic for two hours. Participants appreciated the structure of weekly meetings, valued commitment and follow through, and enjoyed exposure to new ideas. Perhaps most importantly, they valued having a forum where representatives of the healthcare system listened to their experience and their frustration.

## Discussion

Patients at an academic rehabilitation clinic strongly identified with Whole Health as conveyed through the PHI and the “Taking Charge of My Life and Health” course. The model clearly articulated a vision of health promotion as opposed to disease treatment, and patients understood they were the drivers in this “person-driven care”^13^ model.

Open-ended questions about joy, meaning in life, and future visions of self, prompted reflective responses. These types of reflections are essential to person-driven care. The VA describes WH as an opportunity to “change the conversation” from “What is the matter?” to “What Matters.”^6^ The themes that arose in this study such as the centrality of family and friends, the need for independence and autonomy, the power of spiritual connections, and the importance of helping others suggest that the PHI would be successful at broadening the patient-provider conversation.

High attendance in the 10-week course reflected participants’ eagerness for further engagement, and focus group findings helped explain why half of participants were unsure about promoting the course. They were not averse to the self-care strategies promoted in the course, but they also wanted access to the system of care depicted by the model. In this manner, WH materials accentuated the shortcomings of the current healthcare system. Participants raised concerns that their experience of healthcare has been fragmented and confusing.

Participants accurately pointed out that few CIH offerings were available or covered by insurance within this healthcare system at the time of the study. One limitation of the study is that patterns of CIH use among participants were not assessed. Another key limitation is the small sample size. It is hard to generalize results from thirty participants, six of whom completed the course. However, the results presented here contribute to a growing body of knowledge about WH interventions. Further research is needed to understand what is universal and what is specific to this setting and this cohort’s dynamic; another limitation is that training in facilitation of “Taking Charge of My Life and Health” was not available to non-VA staff during the time period of this study. The facilitator (CL) has a Master’s in Public Health specializing in Health Education and experience facilitating groups, as well as research experience in CIH. However, she is not specifically trained in WH or certified in a CIH modality. To strengthen reproducibility, she closely followed the facilitator’s guide (14). Recruitment took place at the same location as a weight management clinic, however there were multiple other physiatrists working out of the same clinic. Therefore, while it is possible that the study sample was skewed toward patients seeking weight management, this was not the only population represented in this study.

## Conclusion

The Whole Health System is the VA’s roadmap for cultural transformation. It communicates their vision for population based person-driven care. At this academic rehabilitation clinic, patients resonated with the Whole Health model as conveyed through the Personal Health Inventory and the “Taking Charge of My Life and Health” course. Findings suggest that the Personal Health Inventory can help change the conversation from “What is the matter?” to “What matters.” Patients were not averse to the self-care strategies promoted in the course, but they also wanted to experience the system of care depicted by the model. They wanted healthcare services that were well coordinated, accessible and personalized. They were eager to share their experiences and to re-imagine the delivery of healthcare in a coordinated and person-driven manner.

## References

[1] Krejci LP Carter K Gaudet T . Whole health: The vision and implementation of personalized. Med Care. 2014;52(12 suppl 5):S5-S8. doi:10.1097/MLR.0000000000000226. 25397823

[2] Vanderweele TJ . On the promotion of human flourishing. Proc Natl Acad Sci Unit States Am. 2017;114(31):8148-8156. doi:10.1073/pnas.1702996114. PMC5547610 28705870

[3] Whole Health Library Home . https://www.va.gov/wholehealthlibrary/. https://www.va.gov/wholehealthlibrary/Accessed July 30, 2021.

[4] Office of patient centered care & cultural transformation (OPCC&CT)–patient care services. https://www.patientcare.va.gov/Patient_Centered_Care.asp. Accessed July 24, 2021.

[5] Comprehensive Addiction and Recovery Act of 2016 (2016; 114th Congress S. 524)–GovTrack.us. https://www.govtrack.us/congress/bills/114/s524. Accessed February 7, 2021.

[6] Kligler B Cotter A Roca H Saenger M . Whole health/integrative health in the VHA: Focusing on what matters to the veteran rather than what is the matter with them. Explore. 2017;13(4):274-276. doi:10.1016/j.explore.2017.04.015. 28651897

[7] Bokhour BG Hyde J Zeliadt S Mohr D . whole health system of care evaluation-a progress report on outcomes of the WHS Pilot at 18 flagship Sites VA center for evaluating patient-centered care in VA (EPCC-VA). 2020. https://www.va.gov/WHOLEHEALTH/professional-resources/clinician-tools/Evidence-Based-. https://www.va.gov/WHOLEHEALTH/professional-resources/clinician-tools/Evidence-Based-Accessed November 20, 2020.

[8] Johnson RB Onwuegbuzie AJ . Mixed methods research: A research paradigm whose time has come. Educ Res. 2004;33(7):14-26. doi:10.3102/0013189X033007014.

[9] Bokhour BG Hogan TP Volkman JE . The personal health inventory: An analysis of veteran responses center for evaluating patient-centered care in VA (EPCC-VA). 2013. Published online: https://www.va.gov/PATIENTCENTEREDCARE/docs/PHIWhitePaperEPCC_12-31-13_508.pdf Accessed June 13, 2018.

[10] Take charge of my life and health–facilitator training, train the trainer–whole health library. https://www.va.gov/WHOLEHEALTHLIBRARY/courses/take-charge-of-my-life-and-health-train-the-trainer.asp. Accessed July 24, 2021.

[11] Braun V Clarke V . Using thematic analysis in psychology. Qual Res Psychol. 2006;3(2):77-101. doi:10.1191/1478088706qp063oa.

[12] U.S. Census Bureau . QuickFacts: North Carolina. https://www.census.gov/quickfacts/NC? https://www.census.gov/quickfacts/NC? Accessed July 30, 2021.

[13] Gaudet T Kligler B . Whole health in the whole system of the veterans administration: How will we know we have reached this future state? J Alternative Compl Med. 2019;25(S1):S7-S11. doi:10.1089/acm.2018.29061.gau. 30870023

[14] Taking Charge of My Live and Health-FT TTT Guide 3-11-2020. https://www.va.gov/WHOLEHEALTHLIBRARY/docs/TCMLH-FT-TTT-Guide.pdf. Accessed December 3, 2021.

